# Comparative study of combined vitrectomy with phacoemulsification versus vitrectomy alone for primary full-thickness macular hole repair

**DOI:** 10.1186/s12886-021-01918-2

**Published:** 2021-04-10

**Authors:** Christophe Valmaggia, Filip Kostadinov, Corina Lang, Josef Guber

**Affiliations:** 1 Department of Ophthalmology, Cantonal Hospital St. Gallen, Rorschacher Strasse 95, 9007 St. Gallen, Switzerland; 2 Department of Otolaryngology, Cantonal Hospital Luzern, Spitalstrasse 6000 Luzern 16, Luzern, Switzerland

**Keywords:** Macular hole, Pars plana vitrectomy, Phacoemulsification, Phacovitrectomy

## Abstract

**Background:**

To assess the effectiveness and safety of 23-gauge pars plana vitrectomy combined with phacoemulsification versus vitrectomy alone in patients over 50 years with primary full-thickness macular holes (FTMH).

**Methods:**

We retrospectively reviewed the medical records related to 406 consecutive vitrectomies performed for primary FTMH. Phacovitrectomy was performed in 294 phakic eyes whereas vitrectomy alone in 112 pseudophakic eyes. The cases were divided into three groups according to the stage of the FTMH: stage 2 (n = 93), stage 3 (n = 270), or stage 4 (n = 43). The primary outcome measure was the closure of the FTMH. The secondary outcome measures were the evolution of visual acuity as well as intraoperative and postoperative complications.

**Results:**

Neither the primary nor the secondary outcomes differed between phacovitrectomy and vitrectomy alone for all three stages. The FTMH were closed in 375 eyes (92.4 %) after a first operation. The closure rate was higher for stage 2 (96.8 %) than for stages 3 (91.1 %) or 4 (90.75 %), but not significantly (P = 0.189). The mean visual acuity increased significantly from preoperatively LogMAR 0.68 (± SD 0.2) to LogMAR 0.43 (± SD 0.24) at the end of the follow-up (p < 0.001).

**Conclusions:**

Combined 23-gauge pars plana vitrectomy with phacoemulsification for primary FTMH repair in patients over 50 years is as efficient and safe when compared with vitrectomy only.

**Trial registration:**

The study was approved on 30^th^ April 2020 by the local ethics committee (Ethikkommission Ostschweiz, EKOS 20/074; BASEC Nr. 2020-01033).

**Supplementary Information:**

The online version contains supplementary material available at 10.1186/s12886-021-01918-2.

## Background

Full-thickness macular hole (FTMH) is defined as a foveal lesion with interruption of all retinal layers from the internal limiting membrane to the retinal pigment epithelium [1]. Tangential or perpendicular traction to the vitreoretinal interface plays an important role in the development of primary FTMH. If the posterior vitreous detachment is incomplete, epiretinal cell proliferation and fibrosis are essential parts of the pathogenesis too [2]. Secondary FTMH is related to other pathologic features such as high myopia, macular schisis, macular telangiectasia, wet age-related macular degeneration, surgical or ocular trauma without any preexisting or concurrent vitreomacular traction [1, 3]. Primary FTMH is diagnosed in 86 to 92 % of cases [4]. The reported prevalence is 0.02–0.8 %, with 7.8 new cases per 100’000 population per year [5]. The peak incidence is thought to be in the seventh decade of life [5]. The male-to-female ratio is 1:2 to 1:3.3 [5]. The female predominance in primary FTMH is explained by the postmenopausal drop in the estrogen levels, leading to increased vitreous liquefaction, and thus a higher likelihood for FTMH [6].

The success of surgery in the treatment of primary FTMH has been reported in several studies [7]. The failure rate of primary surgery is less than 10 % but the nonclosure rate for large FTMH may be as high as 44 % [8, 9]. Nevertheless, the optimal treatment for patients with both vitreoretinal and cataract disease is still a matter of debate [10–12]. A combined surgery, hereafter called phacovitrectomy, or a sequential surgery with a vitrectomy followed by a cataract operation both have their pros and cons [13, 14]. Meanwhile, phacovitrectomy is an accepted approach. However, more postoperative refractive prediction errors as well as increased post-operative inflammatory response in patients undergoing phacovitrectomy are reported [15, 16]. To date, recent studies on phacovitrectomy have mostly reviewed inhomogeneous groups of patients with diverse vitreoretinal pathologies, which makes interpretation difficult [17–20]. The present study was designed specifically to assess the effectiveness and safety of a 23-gauge pars plana vitrectomy for primary FTMH repair in patients over 50 years [21]. A group of patients treated by phacovitrectomy is compared with a group of already pseudophakic patients treated only by vitrectomy. This model allows a direct comparison between phacovitrectomy and vitrectomy alone for a well-defined pathology, avoiding the need to take into account possible complications related to a subsequent cataract operation in the control group.

## Materials and Methods

### Subjects

In this retrospective study, the medical history of all consecutive patients over 50 years of age treated exclusively for primary FTMH at the Cantonal Hospital St. Gallen, Switzerland between January 2010 and December 2019 were reviewed. Patients with secondary FTMH were excluded from the study, namely: high myopia, macular schisis, macular telangiectasia, wet age-related macular degeneration, previous pars plana vitrectomy (e.g. for vitreomacular pathologies or retinal detachment), and blunt trauma [1, 3].

The classification of the FTMH was made using optical coherence tomography (OCT) images (Spectralis, Heidelberg, Germany). The staging was defined according to the modified Gass classification system: stage 2 for macular holes of ≤ 400 μm, stage 3 for macular holes of > 400 μm with an attached vitreous body, and stage 4 for macular holes of > 400 μm with posterior vitreous body detachment [22]. The aperture size of the FTMH was measured at the narrowest hole width using the caliper function of the OCT device as a line drawn approximately parallel to the retinal pigment epithelium [23]. The primary outcome measure was the OCT-confirmed closure of the FTMH defined as a flattened and reattached hole rim along the whole circumference of macular hole without exposed retinal pigment epithelium [23]. The secondary outcome measures were the evolution of the best-corrected visual acuity (BCVA), the intraoperative complications (e.g. posterior capsule rupture, retinal tears, vitreous or retinal bleeding), and the postoperative complications (e.g. lens subluxation, iris capture, endophthalmitis, perfusion disorder, retinal or choroidal detachment, intraocular pressure (IOP) decompensation, macular edema). The BCVA was recorded as a Snellen visual acuity and converted to the logarithm of the minimum angle of resolution (LogMAR) for statistical analysis. For the entire data considered, the end of the follow-up coincided with the last entries in the medical history.

### Surgical procedure

The surgeries were performed under general anesthesia by three senior surgeons (C.V., C.L., and J.G.). A standard pars plana vitrectomy with three 23 gauge-valved ports (Stellaris System, Bausch & Lomb, USA), and a wide-angle viewing system (Oculus BIOM 5, Germany) was performed. A posterior vitreous body detachment was induced if not present, and a core and peripheral vitrectomy was done with scleral indentation. The internal limiting membrane and, if present, the epiretinal membrane were stained (MembraneBlue-Dual DORC, The Netherlands), and removed from the macular area with an end-gripping micro forceps. A search for retinal tears was performed with scleral indentation. Any breaks or suspicious areas were treated either with cryotherapy or argon laser retinopexy. At the end of the surgery, fluid-gas exchange was performed and 20 % sulfur hexafluoride was applied as intraocular tamponade. All three sclerotomies were closed with a synthetic, absorbable suture (Vicryl Rapid 8.0, Ethicon, USA). For the phacovitrectomy, the three 23 gauge-valved ports were placed before performing the cataract surgery. Phacoemulsification was carried out through a 2.6 mm sclerocorneal tunnel incision. The capsulorhexis of the anterior capsule was made with its margin covering the edge of a 6.0-mm optic intraocular lens. The residual cortex was removed using irrigation/aspiration cannulas. A foldable one-piece hydrophobic acrylic intraocular lens was implanted in the capsular bag using a preloaded injector system. The postoperative treatment regime consisted of the topical application of tobramycin (0.3 %) and dexamethasone (0.1 %) three times a day for three weeks. No routine anti-ocular hypertensive medication was given. Patients were advised to posture face-down eight hours for three days.

### Statistical analysis

The categorical variables were compared using a Chi-Square test with Yate correction. For the continuous variables, a One-Way Analysis of Variance test was used for between-group comparisons and a two-tailed paired sample t-Test for within-group comparisons. Logistic regression assessed the correlations between various predictors such as gender, age, stage of the FTMH or preoperative BCVA and the postoperative macular hole closure. A *P*-value < 0.05 was considered statistically significant. The sample size of the study allowed the detection of a LogMAR change of ± 0.1 with a power greater than 90 %, considering a LogMAR standard deviation of 0.2 for the preoperative BCVA and a type-I error α = 5 %. All analyses were carried out with the Social Science Statistics (Version 2020).

## Results

A total of 406 eyes were included in the study. A number of 376 patients (234 women, 142 men) were treated monocularly, and 15 patients (11 women, 4 men) bilaterally. The data of the patients treated with phacovitrectomy or vitrectomy alone for macular holes stage 2, 3, and 4 are reported in Table 1. The evolutions of the BCVA and the IOP are given in Table 2 respectively in Table 3.

**Table 1:** Data of the patients treated with phacovitrectomy or vitrectomy alone for macular holes

	Macular holes stage 2			Macular holes stage 3			Macular holes stage 4		
	Phako-PPV	PPV	*P *Value	Phako-PPV	PPV	*P *Value	Phako-PPV	PPV	*P *Value
Number of eyes (n)	72	21		197	73		25	18	
Women / men (n)	52 / 20	13 / 8	0.524*	127 / 70	39 / 34	0.129*	14 / 11	11 / 7	0.982*
Mean age in years for women (±SD)	68.06 (±5.22)	75.46 (±6.19)	<0.001†	66.12 (±6.84)	75.42 (±8.31)	<0.001†	69.14 (±7.79)	72.64 (±4.24)	0.195†
Mean age in years for men (±SD)	68.50 (±7.57)	71.87 (±6.66)	0.281†	69.17 (±6.93)	75.03 (±6.18)	0.007†	66.45 (±13.74)	72.43 (±7.39)	0.309†
Right eye / left eye (n)	40 / 32	8 / 13	0.248*	97 / 100	31/42	0.393*	11 / 14	9 / 9	0.937*
Mean follow-up in days (±SD)	16.23 (±6.06)	14.33 (±4.23)	0.186†	16.70 (±11.59)	17.88 (±12.51)	0.472†	19.6 (±10.05)	20.44 (±9.83)	0.785†
Macula hole closed / open (n)	71 / 1	19 / 2	0.248*	184 / 13	62/11	0.053*	24 / 1	15 / 3	0.379*
Retinopexy / no retinopexy (n)	24 / 48	6 / 15	0.884*	77 / 120	20 / 53	0.102*	5 /20	3 / 15	0.904*

* Chi-Square test with Yate correction; † One-Way Anova for between-group comparisons; *PPV* Pars plana vitrectomy, *SD* Standard deviation.

**Table 2:** Evolution of the best corrected visual acuity for the patients treated with phacovitrectomy or vitrectomy alone for macular holes

	Macular holes stage 2			Macular holes stage 3			Macular holes stage 4		
	Phako-PPV	PPV	*P *Value	Phako-PPV	PPV	*P *Value	Phako-PPV	PPV	*P *Value
Mean BCVA preoperatively in LogMAR (±SD)	0.60 (±0.28)	0.59 (±0.20)	0.918†	0.69 (±0.28)	0.76 (±0.26)	0.132†	0.95 (±0.47)	0.90 (±0.30)	0.800†
Mean BCVA end of follow-up in LogMAR (±SD)	0.39 (±0.22)	0.31 (±0.19)	0.340†	0.46 (±0.22)	0.51 (±0.33)	0.435†	0.45 (±0.17)	0.49 (±0.16)	0.597†
*P *Value: BCVA preoperatively vs. end of follow-up	<0.001‡	0.003‡		<0.001‡	<0.001‡		0.002‡	0.002‡	

† One-Way Anova for between-group comparisons; ‡ two tailed paired sample t-Testfor within-group comparisons. *BCVA* Best corrected visual acuity, *LogMAR* Logarithm of the minimum angle of resolution, *PPV* Pars plana vitrectomy, *SD* Standard deviation.

**Table 3:** Evolution of the intraocular pressure for the patients treated with phacovitrectomy or vitrectomy alone for macular holes

	Macular holes stage 2			Macular holes stage 3			Macular holes stage 4		
	Phako-PPV	PPV	*P *Value	Phako-PPV	PPV	*P *Value	Phako-PPV	PPV	*P *Value
Mean IOP preoperatively in mmHg (±SD)	14.64 (±2.84)	13.71 (±2.49)	0.181†	15.13 (±2.92)	14.85 (±3.12)	0.488†	14.40 (±2.47)	14.11 (±3.31)	0.744†
Mean IOP first postoperative day in mmHg (±SD)	12.72 (±4.06)	12.52 (±5.47)	0.856†	12.95 (±3.80)	13.19 (±3.51)	0.640†	12.40 (±4.17)	12.44 (±5.93)	0.977†
*P *Value: IOP preoperatively vs. first postoperative day	0.002‡	0.330‡		<0.001‡	0.001‡		0.014‡	0.236‡	
Mean IOP end of follow-up in mmHg (±SD)	15.99 (±4.77)	18.24 (±5.78)	0.073†	16.07 (±4.28)	16.49 (±3.43)	0.459†	16.36 (±2.83)	14.89 (±2.54)	0.087†
*P *Value: IOP preoperatively vs. end of follow-up	0.022‡	<0.001‡		0.011‡	0.003‡		0.002‡	0.391‡	

† One-Way Anova for between-group comparisons; ‡ two tailed paired sample t-Testfor within-group comparisons. *IOP* Intraocular pressure, *PPV* Pars plana vitrectomy, *SD* Standard deviation.

The groups of phacovitrectomy and vitrectomy alone were matched for the following preoperative patients’ characteristics: gender, laterality, mean preoperative BCVA, and mean preoperative IOP. The patients with pseudophakic eyes were older in the vitrectomy alone group which was significant for the women with stages 2, or 3 macular holes (*P* < 0.001), and for the men with stage 3 macular holes (*P* = 0.007).

As for the primary outcome measure, the FTMH were closed in 375 eyes (92.4 %) after a first operation. The closure rate was higher for stage 2 (96.8 %) than for stages 3 (91.1 %) or 4 (90.75 %), but not significantly (*P* = 0.189). For the three stages, the closure rate did not differ significantly if a phacovitrectomy or a vitrectomy alone was performed. Better preoperative BCVA was significantly associated with a higher occlusion rate (*p* = 0.010), while the predictors gender (*p* = 0.94) and age (*p* = 0.75) were not.

As for the secondary outcome measures, the mean BCVA statistically improved from LogMAR 0.68 (± SD 0.2) to LogMAR 0.43 (± SD 0.24) at the end of the follow-up (*P* < 0.001). The mean IOP decreased from 14.83 mmHg (± SD 2.92) to 12.87 mmHg (± SD 4.01) at the first postoperative day (*P* < 0.001), and increased to 16.21 mmHg (± SD 4.02) at the end of the follow-up (*P* < 0.001). The outcomes related to the evolution of BCVA and the IOP did not differ significantly between the groups of patients treated with phacovitrectomy or vitrectomy alone. An intraoperative retinopexy was performed in 135 eyes (33.3 %). This rate was indeed higher for stages 2 (32.5 %) and 3 (35.9 %) compared with stage 4 (18.6 %), but not significantly (*P* = 0.079).

The following postoperative complications were treated medically with success: six cases of ocular hypertension after phacovitrectomy (2 %), and four cases after vitrectomy alone (3.6 %); one case of OCT- confirmed macular edema three weeks after phacovitrectomy (0.3 %). The following postoperative complications were treated surgically: one case of iris capture after phacovitrectomy (0.3 %); two cases of lens subluxation after vitrectomy alone (1.8 %); one case of retinal detachment seven months after vitrectomy alone (0.9 %). Two cases of transient vitreous bleeding each after phacovitrectomy (0.7 %) and vitrectomy alone (1.8 %) were observed; they all resolved spontaneously within three weeks showing no increase in IOP; they presumably arose from the sclerotomies, since other perioperative pathologies were excluded. No treatment was possible in one case of partial vein occlusion diagnosed at the first postoperative day after phacovitrectomy (0.7 %) which resulted in irreversible worsening of BCVA. Specifically, no serious inflammatory reactions had been observed.

## Conclusions

Despite the successful treatment of FTMH with vitrectomy, postoperative cataract formation remains an inevitable consequence whose mechanism is not completely understood. The protective role of the vitreous body was supported in clinical studies as transconjunctival small gauge nonvitrecromizing vitreous surgery for epiretinal membrane induced less progression of cataract compared with conventional small gauge vitrectomy [24, 25]. In the absence of the vitreous gel, molecular oxygen from the retinal vasculature may possibly reach the lens easier and then promote oxidative damage of the lens nucleus, an increase in light scattering, and nuclear sclerotic cataract [26]. Cataract develops at a median of about eight months after vitrectomy, and this delay is reduced to about four months median value if the lens is damaged during the vitrectomy [27]. Usually, the majority of the patients require a cataract surgery within two years of vitrectomy [28]. Moreover, a cataract operation in an already vitrectomized eye is challenging due to lack of support of the vitreous body, the instability of the anterior chamber, and the possible development of a miosis during the procedure [29]. Therefore combined phacovitrectomy has gained popularity. A phacovitrectomy firstly enables patients to improve their visual acuity after a single operation without any deterioration due to cataract progression [30], secondly the surgeons to perform the cataract operation under normal conditions and avoid the difficulties that this operation entails for an eye that has already been vitrectomized [31], and thirdly the healthcare system to reduce the costs and burden of sequential surgery [32].

In the present study, the primary endpoint, macular hole closure and the secondary endpoints, the development of BCVA and intraoperative and postoperative complications, were similar in both procedures, the phacovitrectomy and vitrectomy alone for macular holes of stages 2, 3, and 4. The rate of macular hole closure was comparable with the 90 % or more reported in the literature [33]. The reason for failure may have been insufficient gas tamponade, poor patient compliance with prone position, residual epiretinal traction or incomplete removal of posterior hyaloid despite staining, large macular hole of more than 450 μm for which ILM Flap technique could have been considered, or no obvious causes [8, 9, 34].

The mean BCVA improvement of LogMAR 0.25 was identical to the results of further publications after a similar follow-up [35, 36]. Previous publications about FTMH have reported better surgical and functional results in association with better preoperative BCVA, earlier stage of the disease, younger patient age, and shorter duration of the symptoms [37, 38]. In the present study, a significant correlation was measured only between better preoperative BCVA and higher closure rate (*p* = 0.010). The closure rate trended higher in stage 2 than for stage 3 or 4, but not significantly (*p *= 0.189). Presumably, the data of these three groups did not allow measuring a significant difference between them considering a quite similar high rate of postoperative closure varying between 96.8 and 90.75 % and possibly an insufficient number of patients. The age of the patients did also not correlate with the closure rate (*p* = 0.75). Supposedly, the exclusion of patients younger than 50 years hindered the observation of such a difference. Finally, the duration of symptoms was not reliably recorded in this retrospective study so that this parameter could not be considered.

The complications described in the study are consistent with the literature [5]. Only the retinopexy rate of 33.3 % was higher than the published data of 16.1 % [39]. The retrospective character of our study may explain this discrepancy, as we were no longer able to differentiate whether retinopexy was performed because of intraoperative iatrogenic breaks or for already present retinal degenerations. As expected, the number retinopexies required was lower for stage 4 because of the reduced traction of the prior detached vitreous body in this group. Finally, the frequency of postoperative retinal detachment was lower than the 2.4 % documented in the literature [5].

The main limitation of our retrospective study is the short follow-up in the phacovitrectomy group (mean follow-up 16.83 days, range 5–90 days) as well as in the vitrectomy alone group (mean follow-up 17.61 days, range 5–94 days). Actually, we did not intend to monitor the patients at longer intervals and we referred them back to their private ophthalmologist after the confirmation of closure of the FTMH by the mean of OCT. Although loss to record of complications is unlikely because patients are referred to our regional ophthalmology center not only for surgery but also for long-term issues, we cannot completely exclude the possibility that we have missed some patients and may therefore have underestimated the rate of postoperative complication.

In conclusion, the results of the present study showed similar positive results for a phacovitrectomy in phakic eyes compared to a vitrectomy alone in pseudophakic eyes. According to literature, a combined surgery has several advantages over a sequential surgery [30–32]. Therefore, a 23-gauge PPV with phacoemulsification is a viable option to specifically treat phakic patients over 50 years of age with a primary FTMH.

## Supplementary Information

**Additional file 1.**

## Data Availability

The datasets used and/or analysed during the current study are available from the corresponding author on reasonable request.

## Abbreviations

BCVA: Best-corrected visual acuity
FTMH: Full-thickness macular hole
IOP: Intraocular pressure
LogMar: Logarithm of the minimum angle of resolution
OCT: Optical coherence tomography
