# Supplementary material for: Comparative study of combined vitrectomy with phacoemulsification versus vitrectomy alone for primary full-thickness macular hole repair
Source: BMC Ophthalmol. 2021 Apr 10;21:174. doi: 10.1186/s12886-021-01918-2 (PMC8037829; doi:10.1186/s12886-021-01918-2)
Supplement: Supplementary file 1 — Additional file 1. [file 12886_2021_1918_MOESM1_ESM.pdf]

EKOS
Ethikkommission
Ostschweiz

Ethikkommission Ostschweiz, Scheibenackerstrasse 4, 9000 St.Gallen

Intern
Prof. Dr. med. Christophe Valmaggia
Kantonsspital St. Gallen
Rorschacher Strasse 95
Augenklinik
9007 St. Gallen

Dr. med. Susanne Driessen
Präsidentin

Ethikkommission Ostschweiz
Scheibenackerstrasse 4
9000 St. Gallen
T 058 411 28 91
T 058 411 28 94 Sekretariat
T 058 411 28 93 wissenschaftl. Sekretariat

susanne.driessen@ekos.ch
sekretariat@ekos.ch
https://www.sg.ch/gesundheit-soziales/gesundheit/gremien.html

St. Gallen, 30.4.2020 SD/kj

Verfügung der Ethikkommission Ostschweiz (EKOS)

**Project-ID**	**2020-01033**	EKOS 20/074
**Projekttitel**	Postoperative Ergebnisse bei der Versorgung von Makulaforamina mittels pars plana Vitrektomie	
**Projektleitung**	Prof. Dr. med. Christophe Valmaggia	
**Sponsor**	Prof. Dr. med. Christophe Valmaggia	
**Zentrum**	Prof. Dr. med. Christophe Valmaggia, Augenklinik Kantonsspital St. Gallen, St. Gallen	

Entscheidverfahren

☐ ordentliches Verfahren ☒ vereinfachtes Verfahren ☐ Präsidialverfahren

Entscheid

☒ Die Bewilligung wird erteilt
☐ Die Bewilligung wird mit Auflagen erteilt
☐ Die Bewilligung kann noch nicht erteilt werden
☐ Die Bewilligung wird nicht erteilt
☐ Auf das Gesuch wird nicht eingetreten

Anmerkung:

Auf Erfahrung heraus empfehlen wir, das biostatistische Auswertungskonzept vor der Datensammlung und Erstellung eines entsprechenden Erfassungsbogens mit einem Statistiker zu besprechen.

Klassifizierung

☒ Forschungsprojekt gemäss HFV

Kategorie: --

- ☐ Forschung mit Personen
- ☒ Weiterverwendung des biologischen Materials oder der gesundheitsbezogenen Personendaten
- ☐ mit Verstorbenen
- ☐ mit Embryonen / Föten
- ☐ mit ionisierender Strahlung

☒ Das Forschungsprojekt ist eine Weiterverwendung biologischen Materials und gesundheitsbezogener Personendaten bei fehlender Einwilligung (Art. 34 HFG, Art. 37-40 HFV)

a. **Zweck der Weiterverwendung:**

Es wird der klinische Verlaufs nach pars plana Vitrektomie bei Patienten mit einem Makulaforamen retrospektiv untersucht.

b. **Bezeichnung des biologischen Materials/der gesundheitsbezogenen Personendaten:**

Es werden alle Patienten mit einem Makulaforamen retrospektiv eingeschlossen, welche zwischen 2010 und 2019 in der Augenklinik des KSSG mittels pars plana Vitrektomie operiert worden sind. Wir rechnen mit ca. 400 Patienten.

c. **Personen, die berechtigt sind biologisches Material und gesundheitsbezogenen Personendaten weiterzugeben:**

Prof. Christophe Valmaggia, Chefarzt Augenklinik, KSSG, St. Gallen

d. **Personen, die berechtigt sind biologisches Material und gesundheitsbezogene Personendaten entgegenzunehmen:**

Prof. Dr. med. Christophe Valmaggia
Kantonsspital St. Gallen
Rorschacher Strasse 95 Augenklinik KSSG
9007-St. Gallen
+41 71 4847222
christophe.valmaggia@kssg.ch

Am Entscheid beteiligte Kommissionsmitglieder siehe Anhang.

Die Ethikkommission bestätigt, dass sie nach ICH-GCP arbeitet.

Gebühren

Betrag: CHF 800.-- **Tarifcode: 2.0.**

Gemäss der geltenden Gebührenordnung von swissethics.

Rechtsmittelbelehrung

Gegen einen Beschluss mit Hauptprüfer im Kanton St. Gallen kann **innert 14 Tagen**, von der Zustellung an gerechnet, beim Gesundheitsdepartement des Kantons St.Gallen, Rechtsdienst, Oberer Graben 32, 9001 St. Gallen, durch schriftliche Eingabe (in dreifacher Ausfertigung) **Rekurs** erhoben werden. Gegen einen Beschluss mit Hauptprüfer im Kanton Thurgau kann **innert 14 Tagen**, von der Zustellung an gerechnet, beim Departement für Finanzen und Soziales, Generalsekretariat, Regierungsgebäude, 8500 Frauenfeld, durch schriftliche Eingabe (in dreifacher Ausfertigung) **Rekurs** erhoben werden.
Die Rekursschrift muss einen Antrag, eine Darstellung des Sachverhalts und eine Begründung enthalten; sie ist zu unterzeichnen. Der angefochtene Beschluss ist dem Rekurs beizulegen.

Kopie an

☐ BAG

☐ Andere

Dr. med. Gero Drack, MPH
Vizepräsident

Thomas Schulz, Dipl. Biol.
Wissenschaftliches Sekretariat

Anhang: -Pflichten des Sponsors/der Prüfperson oder der Projektleitung
-Mögliche Entscheide und ihre Bedeutung
-Eingereichte Dokumente, Version 1 vom 27.4.2020
-Am Entscheid beteiligte Kommissionsmitglieder

Anhang

Pflichten des Sponsors/der Prüfperson oder der Projektleitung:

Einreichung Dokumente: revidierte Dokumente und neue Dokumente zur Studie/zum Projekt sollen ausschliesslich über das Web-Portal BASEC eingereicht werden, auf der entsprechenden Formularseite des betreffenden Gesuches. Obsolete Dokumente sind dabei zu entfernen und Datums- und Versionsangaben entsprechend zu ergänzen. Die erfolgten Änderungen müssen im Korrekturmodus abgefasst werden und zusätzlich als ‚clean'-Version eingereicht werden. Die Studieninformationen und -einwilligungen, das Protokoll und die Amendments müssen in durchsuchbaren PDF-Dateien eingereicht werden, insbesondere müssen gescannte Dokumente eine Texterkennung durchlaufen haben (OCR). Das unterschriebene und datierte Begleitschreiben muss die Antworten auf eventuell von der EK gestellte Fragen enthalten. Revidierte Dokumente sind auch den weiteren Zulassungsbehörden zuzustellen, sofern diese involviert sind.

Anmerkung: Die zuständige Ethikkommission überprüft im Rahmen des Bewilligungsverfahrens Aufklärungsbogen und Einwilligungserklärung in einer der Amtssprachen Deutsch, Französisch oder Italienisch. Aufklärungsbogen und Einwilligungserklärung in einer anderen Sprache werden von der Ethikkommission lediglich zur Kenntnis genommen. Für die korrekte Übersetzung ist der Sponsor oder die Projektleitung verantwortlich.

Meldepflichten: Die rechtlich bindenden Melde- resp. Bewilligungspflichten an die Ethikkommission für wesentliche Änderungen, einen vorzeitigen Studienabbruch, unerwünschte Ereignisse u.a. sind einzuhalten (Verordnungen des Bundes). Der Abschlussbericht ist spätesten ein Jahr nach Studienende der Ethikkommission einzureichen.

Registrierungspflicht: Der Sponsor muss – falls es sich um einen klinischen Versuch handelt – diesen in einem WHO-Primärregister oder im Register der Nationalen Medizinbibliothek der USA (clinicaltrials.gov) erfassen und anschliessend diese Nummer im BASEC-Portal eingeben. Die Übertragung der erforderlichen Daten in das Swiss National Clinical Trials Portal (SNCTP) kann nach Bewilligung der Ethikkommission und Zustimmung des Gesuchstellers automatisch erfolgen. Die Informationen über den klinischen Versuch sind in beiden Registern öffentlich zugänglich. Zusätzlich veröffentlicht swissethics wenige Informationen wie Titel, Projekttyp oder Leit-Ethikkommission aller durch die kantonalen Ethikkommissionen bewilligten Gesuche auf swissethics.ch (ausser Phase-I-Studien).

Mögliche Entscheide und ihre Bedeutung

Die Bewilligung wird erteilt: Das Vorhaben kann gemäss bewilligtem Forschungsplan und im Rahmen der anwendbaren rechtlichen Bestimmungen durchgeführt werden. Weitere Bewilligungspflichten (Swissmedic/BAG) sind zu beachten

Die Bewilligung wird mit Auflagen erteilt: Das Vorhaben kann gemäss bewilligtem Forschungsplan gestartet werden und im Rahmen der anwendbaren rechtlichen Bestimmungen durchgeführt werden. Die Auflagen sind zu erfüllen und die Gesuchsunterlagen innert 30 Tagen entsprechend anzupassen. Die revidierten Dokumente werden nach Einreichung im Präsidialverfahren geprüft. Weitere Bewilligungspflichten (Swissmedic/BAG) sind zu beachten

Die Bewilligung kann noch nicht erteilt werden: Das Vorhaben kann noch nicht gestartet werden. Die nachfolgenden Bedingungen sind zu erfüllen bzw. die Fragen zu beantworten und die revidierten Dokumente erneut bei der Ethikkommission einzureichen. Die Ethikkommission überprüft die revidierten Dokumente und erteilt die Bewilligung, wenn die Bedingungen erfüllt bzw. die Fragen zufriedenstellend beantwortet sind.

Die Bewilligung wird nicht erteilt: Das Vorhaben kann in der vorliegenden Form nicht durchgeführt werden. Eine Neueinreichung ist möglich.

Auf das Gesuch wird nicht eingetreten: Begründung siehe vorne, z.B. nicht zuständig oder nicht bewilligungspflichtig.

Eingereichte Dokumente für das Hauptzentrum

Prof. Dr. med. Christophe Valmaggia, Augenklinik Kantonsspital St. Gallen, St. Gallen

Dokument	Dok.Datum	Version
**1. Cover Letter**		
ekos-begleitbrief-ppv-mf-25-4-20.pdf	25/04/2020	
**2. Synopsis of the study plan**		
see doc/cat: **4**, page/ref: **4 / 14 Wissenschaftliche Methodik**		
**4. Study plan (protocol), signed and dated**		
ekos-forschungsprojekt-ppv-mf-25-4-20.pdf	25/04/2020	1
**6. Investigator's CV, dated**		
ekos-cv-valmaggia-25-4-20.pdf	25/04/2020	
**14. Information on secure handling of biological material and personal data, and in particular on the storage thereof**		
see doc/cat: **4**, page/ref: **4 / 18 Angaben zur Aufbewahrung**		
**30. Proof of secure and correct coding**		
see doc/cat: **4**, page/ref: **4 / 17 Vorgehen bei unverschlüsselten Daten**		

Am Entscheid beteiligte Kommissionsmitglieder

Für die vorliegende Verfügung im Vereinfachten Verfahren setzte sich die Ethikkommission wie folgt zusammen und war damit beschlussfähig (Art. 6 der OV-HFG vom 20.9.2013):

	Name, Vorname	Berufliche Stellung / Titel	m	f
Vorsitz	Drack Gero	Dr. med., MPH, Vizepräsident	x	☐
Mitglieder	Gehrer-Hug Monika	lic. iur., Richterin, Vizepräsidentin	☐	x
	Schulz Thomas	Dipl. Biol., Wissenschaftliches Sekretariat	x	☐
